# Premotor dorsal white matter integrity for the prediction of upper limb motor impairment after stroke

**DOI:** 10.1038/s41598-019-56334-w

**Published:** 2019-12-23

**Authors:** Leonardo Boccuni, Sarah Meyer, Nicholas D’cruz, Simon S. Kessner, Lucio Marinelli, Carlo Trompetto, André Peeters, Vincent Van Pesch, Thierry Duprez, Stefan Sunaert, Hilde Feys, Vincent Thijs, Alice Nieuwboer, Geert Verheyden

**Affiliations:** 1 KU Leuven – University of Leuven, Department of Rehabilitation Sciences, Leuven, Belgium; 2 University of Genova, Department of Neuroscience, Rehabilitation, Ophthalmology, Genetics, Maternal and Child Health, Genova, Italy; 3 University Medical Center Hamburg-Eppendorf, Department of Neurology, Hamburg, Germany; 4 Cliniques Universitaires Saint-Luc, Department of Neurology, Brussels, Belgium; 5 Cliniques Universitaires Saint-Luc, Department of Radiology, Brussels, Belgium; 6 KU Leuven – University of Leuven, Department of Imaging and Pathology, Leuven, Belgium; 7 Florey Institute of Neuroscience and Mental Health, Stroke Division, Melbourne, Australia; 8 Austin Health, Department of Neurology, Melbourne, Australia

**Keywords:** Brain, Prognostic markers, Stroke

## Abstract

Corticospinal tract integrity after stroke has been widely investigated through the evaluation of fibres descending from the primary motor cortex. However, about half of the corticospinal tract is composed by sub-pathways descending from premotor and parietal areas, to which damage may play a more specific role in motor impairment and recovery, particularly post-stroke. Therefore, the main aim of this study was to investigate lesion load within corticospinal tract sub-pathways as predictors of upper limb motor impairment after stroke. Motor impairment (Fugl-Meyer Upper Extremity score) was evaluated in 27 participants at one week and six months after stroke, together with other clinical and demographic data. Neuroimaging data were obtained within the first week after stroke. Univariate regression analysis indicated that among all neural correlates, lesion load within premotor fibres explained the most variance in motor impairment at six months (R^2^ = 0.44, *p* < 0.001). Multivariable regression analysis resulted in three independent, significant variables explaining motor impairment at six months; Fugl-Meyer Upper Extremity score at one week, premotor dorsal fibre lesion load at one week, and age below or above 70 years (total R^2^ = 0.81; *p* < 0.001). Early examination of premotor dorsal fibre integrity may be a promising biomarker of upper limb motor impairment after stroke.

## Introduction

The corticospinal tract (CST) is a composite pathway originating from several parietofrontal areas which synergistically integrate several important functions such as descending control of afferent inputs; selection, gating, and control of spinal reflexes, excitation and inhibition of motoneurons^1^; as well as motor control, muscle strength and even basic motor activity^2,3^. Most of our knowledge about the CST comes from animal studies, where the effects of selective lesions to descending motor pathways have been investigated for decades^4,5^. By contrast, only a few studies in humans have considered the diverse origins of the CST, or have separately investigated the white matter descending pathways from the primary motor cortex, premotor areas and primary somatosensory cortex^6–8^.

Existing literature has mainly focused on fibres descending from the primary motor cortex as a biomarker of motor recovery. Motor Evoked Potentials (MEPs) from Transcranial Magnetic Stimulation (TMS) over the primary motor cortex have been considered as an index of CST integrity^9^. According to the presence or absence of MEPs, patients may be classified in two groups, i.e. those with CST integrity and expected recovery of about 70% from initial impairment (MEP+), and those with severely compromised CST integrity and expected limited to no recovery (MEP-)^10^. However, some MEP- patients may still regain motor function to a certain extent, thus TMS assessment needs to be combined with other clinical outcomes and demographic data, in order to reduce the risk of misclassifying patients with some potential for recovery^11,12^. Experts in the field have pointed out that the relatively low negative predictive power of MEP is reflective of the main limitation of TMS, which is, the assessment is restricted to the primary motor cortex^12^.

Neuroimaging techniques have been used to investigate cortical and subcortical motor networks. From this work, several fMRI studies have challenged the unique role of the primary motor cortex on motor planning and execution, by showing a distributed pattern of cortical activation during voluntary movements. The range of cortical activation appears to be associated with the level of motor impairment – the more severe motor impairment, the broader the activation pattern^13^. Recent findings suggest that such patterns may be compensatory rather than maladaptive^14^. Besides these functional activation patterns, structural biomarkers such as fractional anisotropy asymmetry index and lesion volume within specific tracts, may allow the assessment of the whole sensorimotor network to refine the prediction of motor outcomes after stroke^12,15,16^. A recent comprehensive analysis investigated the integrity of several sensorimotor structures beyond CST, highlighting that the inclusion of several cortical and subcortical motor areas significantly improved the ability to predict motor recovery of the upper limb after stroke^17^.

The standard method to quantify the integrity of a specific tract is to investigate the extent that the lesion encroaches on the tract (lesion load). This is defined as the overlay between a seed mask of the tract, usually derived by probabilistic fibre tracking from healthy subjects, and individual lesions obtained by diffusion weighted imaging from stroke patients^18^. A common limitation of conventional fibre tracking is that a trade-off has to be made between the probability that voxels belong to a specific tract (probabilistic threshold) and the preservation of tract volumes. When the probabilistic threshold increases, the overlap between tracts is minimized, but less voxels are retained into tract volumes^19^. This was one of the technical issues that restricted the investigation of CST integrity to fibres descending from the primary motor cortex, by focusing the analysis of CST integrity at the level of the posterior limb of the internal capsule, where fibres are more concentrated and probabilistic thresholds are the highest^20^. The Sensorimotor Area Tract Template (SMATT) is a novel white matter atlas that overcomes the above-mentioned limitations by using an innovative thresholding algorithm. This approach allows, for the first time, the objective discrimination of sub-pathways within CST, as descending from six parietofrontal areas: premotor dorsal cortex, premotor ventral cortex, supplementary motor area, pre-supplementary motor area, primary motor cortex, and primary somatosensory cortex^19^. Each of these areas gives unique contributions to motor planning and execution: spatial (premotor dorsal area) and temporal (supplementary and pre-supplementary motor area) components of coordinated muscle activation for reaching movements, the anticipatory shaping of the hand for effective grasping (premotor ventral area), selective and independent muscle activation for the performance of fine out-of-synergy movements (primary motor cortex), and the integration of exteroceptive and proprioceptive input into feedback/feedforward models for motor control (primary somatosensory cortex)^21–27^. Lesion load delineation for each sub-pathway may lead to the identification of specific determinants of motor impairment early after stroke; which could in return be useful for the prediction of motor outcomes at later phases.

In the present longitudinal study, we overlaid SMATT to individual ischemic lesions in a cohort of 27 people after a first stroke, in order to determine the integrity of CST and its sub-pathways. Lesion load within the whole CST and each CST sub-pathway were subsequently analysed with respect to the clinical outcomes collected within the first week and six months post stroke. The main aims were (1) to investigate the association between lesion load in each sub-pathway of the CST and motor impairment within the first week, and (2) to identify whether, at one week, lesion load within a sub-pathway would be an independent biomarker of motor impairment at six months. We hypothesized that several sub-pathways within the CST would be associated with motor impairment both in the acute and chronic phase. Specifically, we hypothesized that the integrity of fibres descending from primary motor cortex, premotor dorsal cortex and supplementary motor area would show consistent associations with motor impairments, given their functional role and concurrent activation during the execution of voluntary movements^28^.

## Methods

For the present study, 33 consecutive patients were recruited from the acute stroke unit of the University Hospitals Leuven (Belgium) and Cliniques Universitaires Saint-Luc, Brussels (Belgium), from October 2012 to September 2014. Six patients did not complete the assessment at six months and were excluded from the analysis. Inclusion criteria were: first-ever clinically-supported and radiologically-defined ischemic stroke; assessment within the first week after stroke onset; presence of motor impairment, as detected by the Fugl-Meyer Upper Extremity assessment (FM-UE)^29^; and sufficient cooperation to perform the assessment. Subjects were excluded if presenting a pre-stroke Barthel Index^30^ score <95 out of 100; other serious neurological conditions with permanent damage such as subdural hematoma, tumour, encephalitis or trauma that present similarly to stroke; any contraindication to MRI, such as presence of pacemaker, implantable cardioverter defibrillator or neurostimulator; serious communication, cognitive or language deficits, which prevented patients from providing informed consent or could interfere with the assessment protocol. All procedures from the present study were performed in accordance with the Helsinki declaration. Ethical approval was obtained for the present experimental protocol by the Medical Ethical Committees of University Hospitals Leuven (Belgium) and Cliniques Universitaires Saint-Luc, Brussels (Belgium). Informed written consent was obtained from all individual participants included in the study.

### Clinical assessment

Subjects were evaluated by one clinical researcher trained in stroke rehabilitation management (SM). Assessments were performed within the first week (between four and seven days after stroke) and at six months. At the first assessment, patient characteristics were collected including age, gender, hand dominance, intervention delay time after symptom onset, stroke severity (National Institutes of Health Stroke Scale, NIHSS)^31^ and lateralization of infarct.

The FM-UE^29^ assesses motor impairment as a whole for the upper extremity including shoulder, elbow, wrist and hand assessment, from reflex activity to voluntary activation. The total score for the FM-UE ranges between 0 and 66^32^. Excellent reliability and validity have been reported for investigating motor impairment^29^.

### Imaging acquisition and probabilistic fibre tracking template

During the first assessment (four to seven days post stroke), patients also underwent imaging standardized MRI protocol and imaging analysis was performed, as reported elsewhere^33^. MRI data of the brain were obtained using a 3 T system (Achieva 3 T®, Philips Healthcare, Best, The Netherlands). Either 3D or 2D fluid-attenuated inversion recovery (FLAIR) data together with DWI and DTI were acquired. Parameter settings for 2D FLAIR sequences were: echo time = 350 ms, repetition time = 4800 ms, inversion time = 1650 ms, matrix = 250 × 250 mm2, slice thickness = 1.12 mm, and spacing between slices = 0.56 mm. For 3D FLAIR sequences: echo time = 422 ms, repetition time = 4800 ms, inversion time = 1650 ms, matrix = 228 × 228 mm^2^, slice thickness = 1.12 mm, flip angle = 90°, spacing between slices = 0.56 mm. Parameter setting for DWI sequences were: number of slices = 58, number of gradient directions = 60, b-value = 0 and 1300 s/mm^2^, echo time = 72 ms, repetition time = 12 s, slice thickness = 2.5 mm, gap = 2.5 mm. Parameter settings for DTI sequences were: number of slices = 182, slice thickness = 1.2 mm, acquisition matrix = 250 × 250 mm^2^, b-values = 0 and 1300 s/mm^2^, number of gradient directions = 60, echo time = 4.6 ms, repetition time = 9.6 ms, spacing between slices = 1.2 mm, flip angle = 8°.

In order to investigate the integrity of specific sensorimotor pathways, individual lesion volumes were overlaid to a sensorimotor area tract template (SMATT), an innovative probabilistic fibre tracking template which segments six corticospinal tracts descending from the primary motor cortex (M1), dorsal premotor cortex (PMD), ventral premotor cortex (PMV), supplementary motor area (SMA), pre-supplementary motor area (PreSMA) and primary somatosensory cortex (S1)^19^. Its unique ability to discriminate several tracts within the pyramidal pathway is due to the use of an algorithm that takes into account a probabilistic threshold to each z-slice (slice level thresholding), thus minimizing overlap between tracts while preserving tract volume^19^. Lesion load was therefore calculated for each sub-pathway separately. Subsequently, a weighting factor was applied to each z-slice, in order to take into account the narrowing of the pyramidal tract, as it descends from parietofrontal sensorimotor areas to the posterior limb of the internal capsule^16,18^. For the present study, lesion load was calculated as follows (adapted from^18^):

$${\rm{w}} \mbox{-} {\rm{PMD}} \mbox{-} {\rm{LL}}=\,\sum (n(x)\times \frac{m({x}^{\ast })}{m(x)}\,)$$

where, for a particular slice (*x*) of a specific tract (for instance, PMD) that contains *n(x*) voxels of lesion; *n(x)* is multiplied by the ratio between m(x^*^), which is the largest slice area (voxels) of PMD; and *m(x)*, which is the area of PMD at the level of slice *x*. Weighted slice lesions were summed together, giving the overall weighted volume of lesion load (in this case, w-PMD-LL) (Part I, supplementary online material).

### Statistical analysis

Patient characteristics were analysed using descriptive statistics. Spearman *ρ* correlation coefficients were calculated between motor impairment at one week and lesion load within the whole CST and each CST sub-pathway, in order to quantify cross-sectional associations between clinical outcomes and each weighted lesion load. Results were interpreted according to Munro’s classification system^34^. Univariate linear regression analysis was further performed between each neural correlate at one week and motor outcome (FM-UE) at six months. Dominance analysis^35^ was performed between neuroimaging predictors, in order to compare their relative importance for the prediction of motor impairment at six months. Bootstrapping was also performed, in order to determine the level of reproducibility of results from dominance analysis across 1000 resamples. The neuroimaging predictor showing dominance over the others, meaning largest additional R^2^ contribution across all regression subset models, was entered into a multivariable linear regression model (stepwise method), together with previous established predictors, namely initial upper limb motor impairment^36^ and age^11^. Tests for model assumptions as well as goodness-of-fit tests were performed. In particular, multicollinearity between predictors was assessed through correlation analysis; presence of outliers by means of casewise diagnostics for standardized residuals; homoscedasticity through visual inspection of plotted standardized predicted values (x-axis) and studentized residuals (y-axis); normally distributed errors by looking at frequency distribution for standardized residuals, normal P-P plots and Kolmogorov-Smirnov test; and independent errors were assessed through Durbin-Watson test. Resampling was performed by means of bootstrapping (single sampling method, 1000 samples, 95% CI), and adjusted R^2^ was considered as parameter for cross-validation. Level of significance (2-tailed) was set at *p* < 0.05. Analyses were performed using IBM SPSS Statistics for Windows, Release 25 (Armonk (NY), USA).

## Results

**Figure 1:**
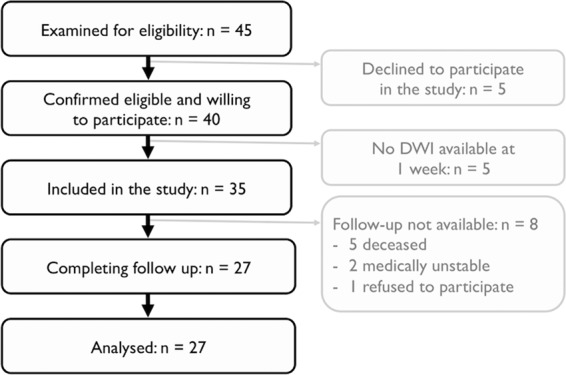
Flow chart of stroke patients included in the analysis.

**Figure 2:**
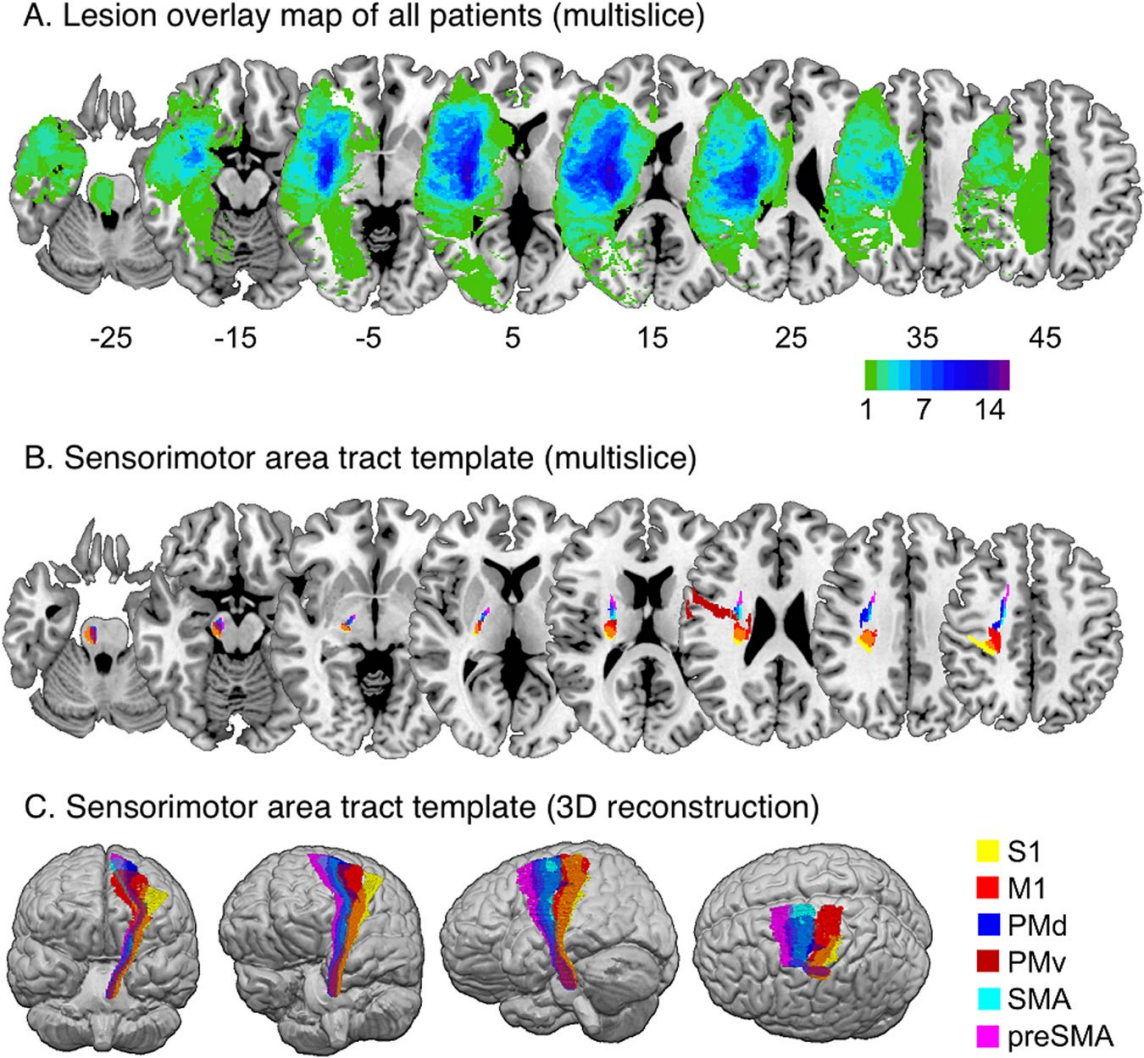
Lesion overlay and white matter template. Individual lesions have been overlaid (multislice, **A**), indicating that most of the lesions involved the middle cerebral artery territory at different levels, where sub-pathways of corticospinal tract are located (multislice and 3D reconstruction, **B** and **C**).

In total, 27 patients were included in the present analysis (Fig. 1). All patients were evaluated at four to seven days (median: 6, IQR: 5–7 days) and at six months (median: 183, IQR: 182–186 days) from stroke onset. Demographic and clinical characteristics are reported in Table 1. Figure 2 gives an overview of the distribution of individual stroke lesions (lesion overlay map) and the mapping of the CST sub-pathways. This shows that most of the lesions involved areas of the middle cerebral artery territory at different levels, where sub-pathways of the corticospinal tract are located.

**Table 1:** Patients characteristics.

Age stroke onset: years, median (IQR)	68 (61–77)
Gender, n (%)	
Male	13 (48)
Female	14 (52)
Days after stroke, median (IQR)	
Four to seven days	6 (5–7)
six months	183 (182–186)
Affected hemisphere, n (%)	
Left	8 (30)
Right	19 (70)
Hand dominance, n (%)	
Left	1 (4)
Right	26 (96)
Stroke severity (NIHSS) within one week, median (IQR)	8 (5–13)
FM-UE within one week, median (IQR)	19 (3–55)
FM-UE at six months, median (IQR)	54 (9–64)

FM-UE indicates Fugl-Meyer Upper Extremity assessment; and NIHSS, National Institutes of Health Stroke Scale.

Results from the correlation analysis are reported in Table 2. Upper limb motor impairment at one week was moderately correlated with the lesion load in the premotor dorsal fibres (w-PMD-LL, *ρ* = −0.53, *p* = 0.004) and supplementary motor area fibres (w-SMA-LL, *ρ* = −0.55, *p* = 0.003), and showed a low correlation with the lesion load in the primary motor fibres (w-M1-LL, *ρ* = −0.48, *p* = 0.012) and the whole CST (w-SMATT-LL, *ρ* = −0.43, *p* = 0.027). Lesion load within PMV, preSMA and S1 were not significantly associated with upper limb motor impairment at one week.

**Table 2:** Associations between upper limb motor impairment at one week and weighted lesion load within CST and CST sub-pathways.

	FM-UE	w-SMATT-LL	w-M1-LL	w-PMD-LL	w-PMV-LL	w-preSMA-LL	w-SMA-LL
w-SMATT-LL	−0.43 (0.027)						
w-M1-LL	−0.48 (0.012)	0.85 (<0.001)					
w-PMD-LL	−0.53 (0.004)	0.89 (<0.001)	0.69 (<0.001)				
w-PMV-LL	−0.36 (0.062)	0.87 (<0.001)	0.70 (<0.001)	0.81 (<0.001)			
w-preSMA-LL	−0.36 (0.064)	0.79 (<0.001)	0.51 (0.007)	0.87 (<0.001)	0.72 (<0.001)		
w-SMA-LL	−0.55 (0.003)	0.89 (<0.001)	0.71 (<0.001)	0.98 (<0.001)	0.81 (<0.001)	0.84 (<0.001)	
w-S1-LL	−0.30 (0.126)	0.81 (<0.001)	0.93 (<0.001)	0.64 (<0.001)	0.64 (<0.001)	0.47 (0.012)	0.63 (<0.001)

Spearman *ρ* correlation coefficients (*p* values, 2-tailed) between weighted lesion load and motor impairment at one week.

FM-UE indicates Fugl-Meyer Upper Extremity assessment; and w-(x)-LL: weighted lesion load within CST (SMATT), and within CST sub-pathways descending from primary motor cortex (M1), premotor dorsal area (PMD), premotor ventral area (PMV), pre-supplementary motor area (preSMA), supplementary motor area (SMA), primary somatosensory cortex (S1).

**Figure 3:**
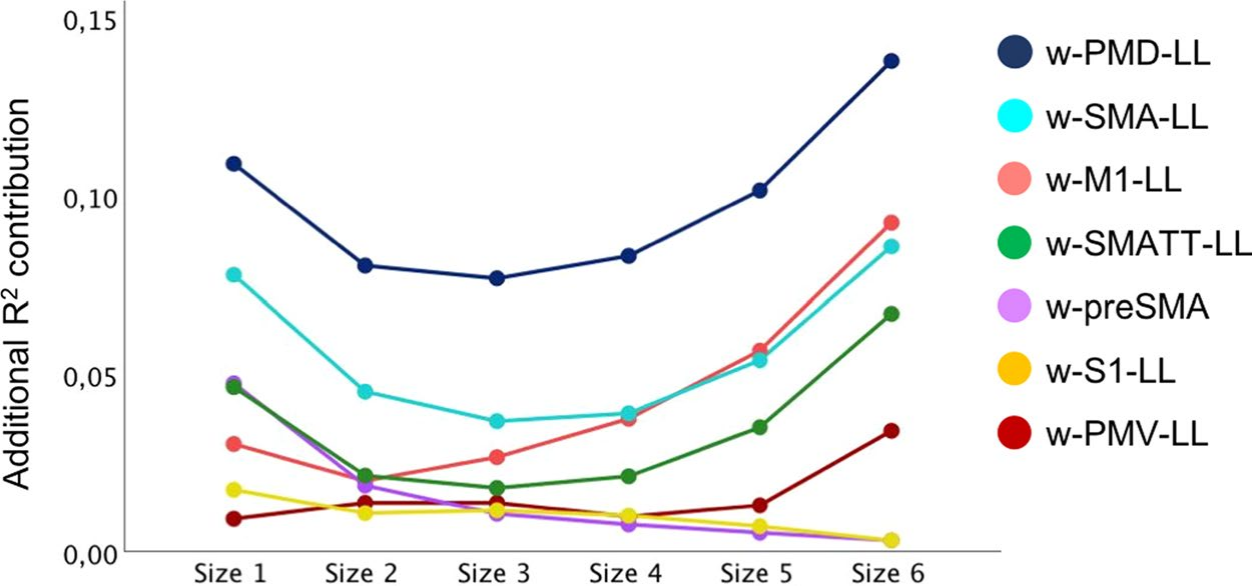
Dominance analysis. Dominance analysis determines the dominance of one predictor over another by comparing their additional R^2^ contributions across all subset models. The different sizes (x-axis) refers to the number of predictors that were included in multivariate regression models, together with the predictor under investigation. The additional contribution of the predictor under investigation is reported as R^2^ increase (y-axis). Therefore, each dot represents the average additional R^2^ contribution of a specific predictor, across multivariate regression models of the same size.

Univariate regression analysis was performed to seek for the best clinical and neuroimaging predictors of motor impairment at six months (Part II, supplementary online material). All weighted lesion load were significantly associated with motor impairment, but they were also moderately to highly intercorrelated. Therefore, dominance analysis was performed to determine the relative importance of a neuroimaging predictor over another. In the original sample, w-PMD-LL showed *complete dominance*^35^ over all other predictors, and across all model subsets (Fig. 3).

However, bootstrapping the dominance analysis revealed a more equitable picture. Complete dominance could not be established for any of the sub-pathways with low reproducibility values. When considering the average additional R^2^ contributions over all subset models (*general dominance*), premotor predictors showed general dominance over all other predictors, with moderate to high levels of reproducibility (reproducibility range = 0.64–0.91 for w-PMD-LL; 0.74–0.93 for w-SMA-LL; 0.88–0.97 for w-PMV-LL). Moreover, w-PMV-LL dominated over w-SMA-LL and w-PMD-LL, and w-SMA-LL dominated over w-PMD-LL (extended output from dominance analysis on part III, Supplementary online material).

**Figure 4:**
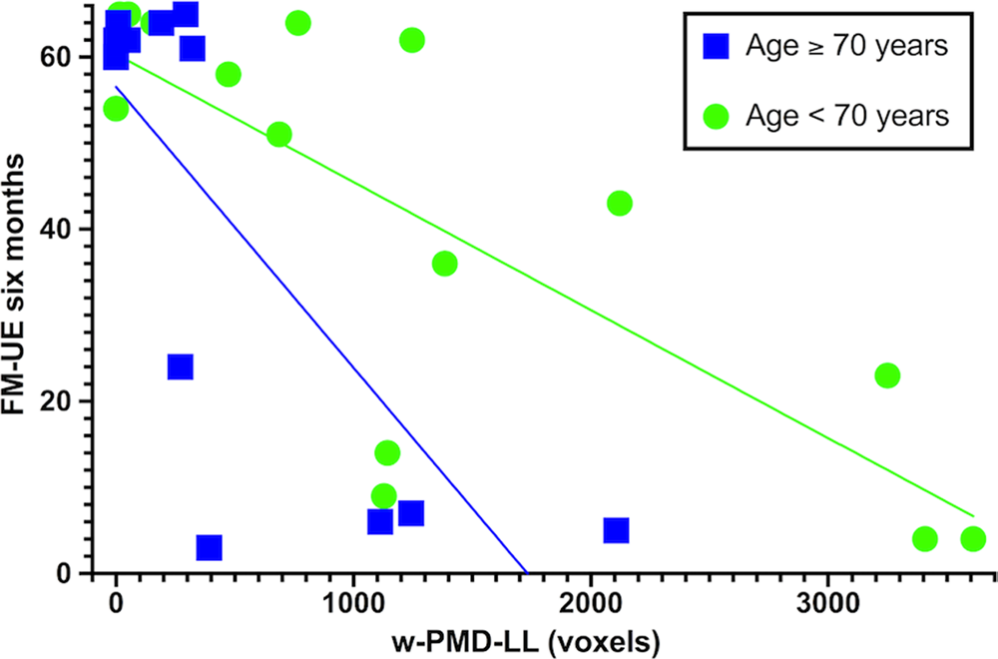
Prediction of upper limb motor impairment at six months. Scatterplot for motor impairment at six months (y-axis) versus weighted lesion load within PMD (x-axis), grouped for age (blue squares: ≥70 years; green dots: <70 years).

To summarize, lesion load in the premotor dorsal cortex (w-PMD-LL) showed, among all neural correlates, the highest explained variance in univariate regression (R^2^ = 0.44, *p* < 0.001); and complete dominance over the other predictors in the original cohort. When all neural correlates where included as predictors in subsets of multivariate regression models, w-PMD-LL showed the highest additional contribution (overall R^2^ = 0.15), a unique association with motor impairment at six months (Squared semipartial correlation = 0.14, *p* = 0.02), and a complete dominance. For these reasons, it was retained as neuroimaging predictor for the multivariable analysis. Besides w-PMD-LL, the FM-UE score at one week and age were retained for the multivariable model (Table 3). Notably, when considering age as a continuous variable in the univariate regression model, results were not significant. However, for patients with the same lesion load, we noticed a difference in age, in that patients older than 70 years showed poorer motor outcomes; therefore, we included age as dichotomous variable (younger or older than 70 years). The resulting regression model (total R^2^ = 0.81, *p* < 0.001) revealed a significant individual contribution of FM-UE (R^2^ = 0.68 *p* < 0.001), w-PMD-LL (R^2^ = 0.07, *p* = 0.021) and age (R^2^ = 0.06, *p* = 0.010), indicating that patients with more severe upper limb motor impairment at one week, greater PMD lesion load and age ≥70 years showed more severe upper limb motor impairment at six months post stroke (Fig. 4). Goodness-of-fit tests confirmed assumptions of regression models; in particular, for multicollinearity assumption, correlations between predictors were far below the threshold of 0.9 and no outlier was detected; assumptions for residuals were tested (homoscedasticity, normally distributed errors, independent errors) with overall positive results. Bootstrapping corroborated the robustness of beta coefficients in the regression model. Finally, cross-validation showed comparable values between adjusted and unadjusted R^2^, thus confirming the generalizability of results from the present sample (Part IV, supplementary online material).

**Table 3:** Multivariate regression analysis for upper limb motor impairment at six months.

Step	Predictors	R	R^2^ (*p*)	Adjusted R^2^	R^2^-change (*p*)	SEE
1	FM-UE one week	0.82	0.68 (<0.001)	0.67	0.68 (<0.001)	14.62
2	FM-UE one week	0.86	0.75 (<0.001)	0.72	0.07 (0.021)	13.33
	w-PMD-LL					
3	FM-UE one week	0.90	0.81 (<0.001)	0.78	0.06 (0.010)	11.78
	w-PMD-LL					
	Age (dichotomized)					

FM-UE indicates Fugl-Meyer Upper Extremity assessment; SEE: Standardized Error of the Estimate; and w-PMD-LL: weighted lesion load within CST sub-pathway descending from premotor dorsal area.

## Discussion

The present study investigated the associations between CST sub-pathways at one week and motor impairment of the upper limb at one week and at six months after stroke. A recently proposed and innovative white matter template enabling the discrimination of sub-pathways within CST was used. This is the first study to implement this template in a cohort of stroke patients, and results indicated that (1) at one week after stroke, motor impairment showed moderate correlations with the integrity of fibres from the PMD and SMA sub-pathways, and low correlations with the integrity of fibres from M1 and the CST taken as a whole; and (2) PMD lesion load at one week was the most dominant neuroimaging predictor of upper limb motor impairment at six months, and along with initial motor impairment and age was able to explain a large amount of variance in six-month upper extremity Fugl-Meyer scores.

In the acute phase, only lesions within M1, SMA and PMD were significantly associated with motor impairment. Such results are confirmative of the specific association of different parts of the corticospinal tract with motor impairment and recovery^10,16^. M1, SMA and PMD differ in a way that each of them receives a unique pattern of input from the parietal lobe, as well as from subcortical motor centres^37^. In non-human primates, tracts descending from PMD and SMA project to the spinal cord directly and through corticobulbar projections to the reticulospinal tract, mainly ipsilaterally^38^. Conversely, corticospinal and corticobulbar fibres from M1 are mainly contralateral, and the corticobulbar tract is less dense compared to the sub-pathways from premotor areas^38^. Premotor areas are reciprocally interconnected with M1^39,40^, and contribute mainly to spatial (PMD) and temporal (SMA) components of internally generated sequential movements of the upper limb^23,24^. Each fibre descending from M1 makes contact with few motor nuclei in the spinal cord, thereby allowing fine and selective control over a small group of muscles, which is required for independent movements of the fingers^41,42^. Besides such differences, premotor areas and primary motor cortex show a comparable number of corticospinal fibres and a concurrent activation during the execution of simple and complex voluntary movements, suggesting that their contribution to motor function is a matter of gradients rather than exclusive specific contributions^37,43^. Accordingly, our results confirm that disruption of white matter underlying PMD, SMA and M1 are associated with upper limb motor impairment.

We further investigated early predictors of upper limb motor impairment at six months. We entered into a multivariate regression model well-known clinical predictors such as initial upper limb motor impairment^36^ and age^11^, together with lesion load within PMD, as it showed the largest univariate explained variance of motor impairment, and a complete dominance over other neuroimaging predictors. Results displayed a significant contribution of each variable to upper limb motor outcome, with an overall 81% of explained variance for motor impairment at six months. In particular, we observed that patients aged above 70 years displayed relatively worse outcome, which partly explained the difference in clinical outcome between patients with comparable lesion load. Our findings suggest that upper limb motor impairment at six months is largely based on a combination of integrity of the PMD corticospinal sub-pathway, initial clinical upper limb impairment and age, which is in line with well-established algorithms for early prediction of upper limb motor impairment^11,44^. Several functional connectivity and cortical stimulation studies suggested that premotor areas may subserve motor recovery post stroke, especially for patients with more severe impairment^45–48^.

Some limitations need to be addressed. For the original cohort being investigated, there was a clear pattern of dominance for w-PMD-LL over the other neuroimaging predictors. However, bootstrapping of dominance analysis showed that other premotor pathways, such as those descending from SMA and PMV, were dominant over PMD across resamples. Reproducibility is an important issue to infer the dominance of a predictor over another beyond the original sample. Notably, bootstrapping indicated that premotor pathways, specifically PMD, PMV and SMA, were always dominant over primary sensorimotor pathways (M1, S1) as well as the CST when taken as a whole. Future, larger cohort studies might be diriment for the determination of specific CST sub-pathways of interest. Also, there is indeed a degree of overlap between CST sub-pathways, in that some voxels are accounted to more than one tract. The novelty of SMATT however, is this balanced compromise between preservation of tract volume (sensitivity) and probability that a voxel belongs to a specific tract (specificity). Nevertheless, this overlap remains an important issue, which should be realized when considering lesion load as predictor. We applied the white matter template as it was originally released, as it uses an innovative algorithm for axial slice-by-slice tract delineation. Further improvements may consider a probabilistic threshold for each voxel to belong to a specific tract, similarly to what has been done for the delineation of stroke lesions (voxel-by-voxel lesion likelihood)^17^. Finally, other potential contributors of clinical impairment, such as lesion load within basal ganglia and reticulospinal tract were not measured, as we had not enough patients with lesions in those neural structures. However, our sample covered a broad range of stroke lesions and clinical outcomes, and we believe to have included a representatively impaired sample of the general stroke population. Despite such heterogeneity, our results indicate that PMD lesion load evaluation could be a potential alternative to M1 for early prediction of upper limb motor impairment at six months after stroke.

## Supplementary information

Supplemental material

## Data Availability

The data that support the findings of this study are available from the corresponding author upon reasonable request
