# Supplementary material for: Premotor dorsal white matter integrity for the prediction of upper limb motor impairment after stroke
Source: Sci Rep. 2019 Dec 23;9:19712. doi: 10.1038/s41598-019-56334-w (PMC6928144; doi:10.1038/s41598-019-56334-w)
Supplement: Supplementary file 1 — Supplemental material [file 41598_2019_56334_MOESM1_ESM.pdf]

SUPPLEMENTARY MATERIAL

Premotor dorsal white matter integrity for the prediction of upper limb motor impairment after stroke

Leonardo Boccuni, BSc[1,2]; Sarah Meyer, PhD[1]; Nicholas D'cruz, MSc[1]; Simon S. Kessner, MD[3]; Lucio Marinelli, MD, PhD[2]; Carlo Trompetto, MD, PhD[2]; André Peeters, MD[4]; Vincent Van Pesch, MD, PhD[4]; Thierry Duprez, MD[5]; Stefan Sunaert, MD, PhD[6]; Hilde Feys, PhD[1]; Vincent Thijs, MD, PhD[7,8]; Alice Nieuwboer, PhD[1]; Geert Verheyden, PhD[1]

1 KU Leuven, Department of Rehabilitation Sciences, Leuven, Belgium

2 University of Genova, Department of Neuroscience, Genova, Italy

3 University Medical Center Hamburg-Eppendorf, Department of Neurology, Hamburg, Germany

4 Cliniques Universitaires Saint-Luc, Department of Neurology, Brussels, Belgium

5 Cliniques Universitaires Saint-Luc, Department of Radiology, Brussels, Belgium

6 KU Leuven, Department of Imaging and Pathology, Leuven, Belgium

7 Florey Institute of Neuroscience and Mental Health, Stroke Division, Melbourne, Australia

8 Austin Health, Department of Neurology, Melbourne, Australia

List of contents:

Part I. How to calculate lesion load

Part II. Univariate regression analysis

Part III. Dominance analysis

Part IV. Goodness of fit test for multivariable regression

Part V. Differences in weighted lesion load within CST sub-pathways

Part I. How to calculate lesion load

Online Table I. Script for lesion load

Step		Code	Notes
1	Place tract images and subject images in the same folder		
2	Create text file with all tracts and subjects	ls -1 *.nii > images.txt	lists all nifti files in directory and outputs to a text file
3	Create array of subject IDs	subj=(sub001 sub002 sub003 sub004 sub005 sub006 sub007 sub008)	This can be copied from the text file (after removing the extensions)
4	Create array of tract names	voi=(10_SMATT 11_M1 12_preSMA 13_SMA 14_PMD 15_PMV 1_TCT 2_S1 3_INSULA 4_BG 5_Caudate 6_Putamen 7_GPi 8_GPe 9_STN)	Similar to previous step. An easy way to do this is to open the images.txt file in Excel and replace the string .nii
5	Nested loop to intersect tracts and subject lesions and output number of overlapping voxels	for i in {0..14} ; do for j in {0..7} ; do fslstats ./${subj[j]}.nii -k ./${voi[i]}.nii -V >> ./${voi[i]}.txt ; done ; done	Adapt brace expansions for i (number of tracts), j (number of subjects). Output is one text file for each tract, with each subject value on new line

Scripts are available for UNIX systems using BASH shell and FSL software

Online Table II. Script for weighted lesion load

Step		Code	Notes
1	Place tract images and subject images in the same folder		
2	Create text file with all tracts and subjects	ls -1 *.nii > images.txt	lists all nifti files in directory and outputs to a text file
3	While loop to slice all images along the z axis	while read p ; do mkdir ./Results/${p:0: -4} ; fslslice $p ./Results/${p:0: -4}/${p:0: -4} ; done < images.txt	reads text file and for each line performs image slicing and outputs to a separate folder for each image
4	While loop to extract number of non-zero voxels per slice (tracts and subjects)	while read p ; do for i in {0000..0181}; do fslstats ./Results/${p:0: -4}/${p:0: -4}_slice_${i}.nii -V >> ./Results/${p:0: -4}/${p:0: -4}.txt ; done ; done < images.txt	Number in brace expansion should be adapted for number of Z slices in the images, here Z = 182
5	Create array of subject IDs	subj=(sub001 sub002 sub003 sub004 sub005 sub006 sub007 sub008)	This can be copied from the text file (after removing the extensions)
6	Create array of tract names	voi=(Right-M1-S-MATT Right-PMd-S-MATT Right-PMv-S-MATT Right-preSMA-S-MATT Right-S1-S-MATT Right-SMA-S-MATT S-MATT)	Similar to previous step. An easy way to do this is to open the images.txt file in Excel and replace the string .nii
7	Nested loop to intersect corresponding slices of tract and subjects lesion and output number of overlapping voxelsoutput number of overlapping voxels	for i in {0..7} ; do for j in {0..6}; do for k in {0000..0181} ; do fslstats ./Results/${subj[i]}/${subj[i]}_slice_$k.nii -k ./Results/${voi[j]}/${voi[j]}_slice_$k.nii -V >> ./Results/${subj[i]}/${voi[j]}.txt ; done ; done ; done	Adapt brace expansions for i (number of subjects), j (number of tracts), k (number of Z slices). Output is one text file for each subject for each region with each slice value on a new line.

Scripts are available for UNIX systems using BASH shell and FSL software

Part II. Univariate regression analysis

Online Table III. Univariate regression analysis

Univariate predictor	R	R^2	Adjusted R^2	SEE	*p*
FM-UE one week	.82	.68	.67	14.62	<.001
Age (dichotomized)	.01	.00	-.04	25.87	.936
w-SMATT-LL	.60	.37	.34	20.60	.001
w-PMD-LL	.66	.44	.41	19.44	<.001
w-SMA-LL	.64	.40	.38	19.97	<.001
w-preSMA-LL	.60	.36	.33	20.72	.001
w-M1-LL	.57	.33	.30	21.20	.002
w-S1-LL	.54	.29	.26	21.81	.004
w-PMV-LL	.50	.25	.22	22.44	.008

FM-UE indicates Fugl-Meyer Upper Extremity assessment; SEE: Standardized Error of the Estimate; w-(x)-LL: weighted lesion load within CST sub-pathways descending from primary motor cortex (M1), premotor dorsal area (PMD), premotor ventral area (PMV), pre-supplementary motor area (preSMA), supplementary motor area (SMA) and primary somatosensory cortex (S1).

Online Figure I. Scatterplot representing univariate regression analysis

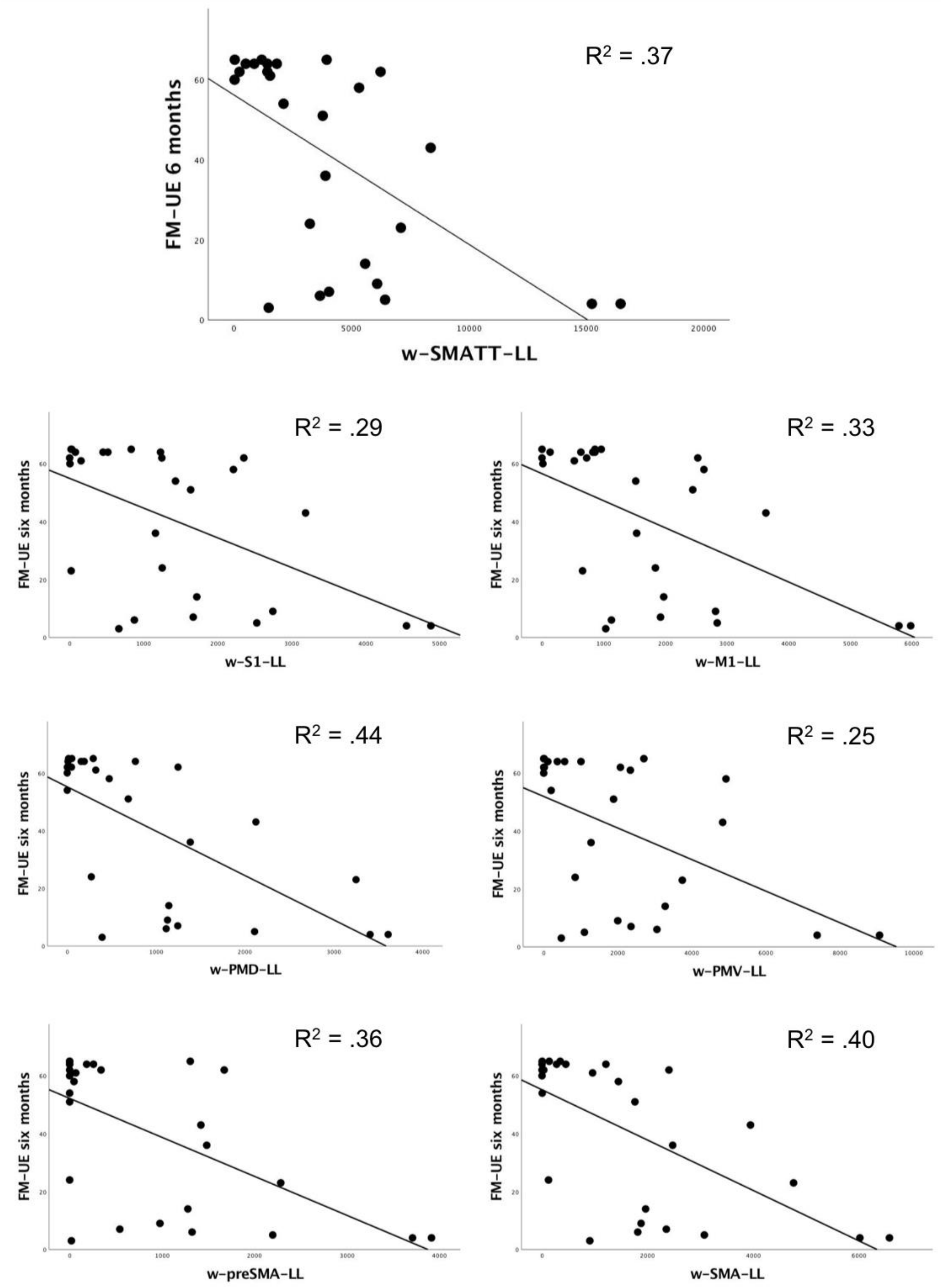

Scatterplots representing univariate regression analyses for the prediction of motor impairment at six months post stroke (y-axis), with weighted lesion load of each sub-pathway within CST as independent variable (x-axis).

Part III. Dominance analysis

Main dominance analysis output (as from https://sites.uwm.edu/azen/damacros/).

Note: data relevant for the present study have been highlighted in yellow.

Variable	DF	Parameter Estimate	SE	t	Pr > |t|	Standardized Estimate	Squared Semi-partial Corr Type II	Squared Partial Corr Type II
Intercept	1	60.49685	5.81552	10.40	<.0001	0	.	.
w_SMATT	1	0.01950	0.01117	1.74	0.0972	3.16276	0.06687	0.13809
w_PMD	1	-0.09523	0.03799	-2.51	0.0214	-4.08458	0.13804	0.24853
w_PMV	1	-0.00859	0.00690	-1.25	0.2279	-0.78491	0.03411	0.07554
w_M1	1	-0.03778	0.01841	-2.05	0.0542	-2.31270	0.09252	0.18146
w_preSMA	1	-0.00492	0.01288	-0.38	0.7066	-0.21873	0.00321	0.00763
w_SMA	1	0.04114	0.02081	1.98	0.0628	3.00428	0.08585	0.17060
w_S1	1	0.00484	0.01277	0.38	0.7088	0.25379	0.00316	0.00751

IN	RSQ	CP2	CP3	CP4	CP5	CP6	CP7		
**0**	**.0000**	.4357	.2476	.3284	.3585	.4043	.2897		
**1**	**.3660**	.0698	.0239	.0005	.0129	.0394	.0000		
**1**	**.4357**	.	.0040	.0125	.0015	.0073	.0098		
**1**	**.2476**	.1920	.	.0822	.1119	.1657	.0586		
**1**	**.3284**	.1198	.0015	.	.0632	.0848	.0065		
**1**	**.3585**	.0787	.0010	.0331	.	.0484	.0233		
**1**	**.4043**	.0387	.0090	.0089	.0027	.	.0068		
**1**	**.2897**	.1557	.0165	.0452	.0921	.1214	.		
**2**	**.4358**	.	.0123	.0557	.0024	.0116	.0281		
**2**	**.3900**	.0582	.	.0002	.0057	.0391	.0031		
**2**	**.3665**	.1250	.0236	.	.0280	.0535	.0024		
**2**	**.3790**	.0593	.0167	.0155	.	.0281	.0037		
**2**	**.4054**	.0421	.0237	.0146	.0017	.	.0083		
**2**	**.3661**	.0979	.0270	.0028	.0165	.0476	.		
**2**	**.4397**	.	.	.0323	.0007	.0035	.0216		
**2**	**.4482**	.	.0237	.	.0044	.0391	.0004		
**2**	**.4372**	.	.0031	.0154	.	.0083	.0125		
**2**	**.4430**	.	.0002	.0443	.0025	.	.0286		
**2**	**.4454**	.	.0158	.0032	.0042	.0262	.		
**2**	**.3299**	.1420	.	.	.0696	.1065	.0053		
**2**	**.3595**	.0808	.	.0399	.	.0575	.0243		
**2**	**.4133**	.0299	.	.0230	.0036	.	.0155		
**2**	**.3062**	.1550	.	.0290	.0776	.1226	.		
**2**	**.3916**	.0610	.0078	.	.	.0235	.0031		
**2**	**.4132**	.0742	.0232	.	.0020	.	.0003		
**2**	**.3349**	.1137	.0003	.	.0598	.0786	.		
**2**	**.4069**	.0385	.0100	.0082	.	.	.0059		
**2**	**.3818**	.0678	.0021	.0129	.	.0311	.		
**2**	**.4111**	.0606	.0177	.0024	.0018	.	.		
**3**	**.4481**	.	.	.0435	.0055	.0050	.0170		
**3**	**.4915**	.	.0002	.	.0039	.0499	.0008		
**3**	**.4383**	.	.0154	.0571	.	.0218	.0257		
**3**	**.4475**	.	.0056	.0939	.0126	.	.0364		

**3**	**.4639**	.	.0012	.0283	.0000	.0199	.		
**3**	**.3901**	.1015	.	.	.0098	.0463	.0072		
**3**	**.3956**	.0580	.	.0043	.	.0335	.0001		
**3**	**.4291**	.0240	.	.0073	.0000	.	.0018		
**3**	**.3931**	.0721	.	.0043	.0026	.0378	.		
**3**	**.3944**	.1009	.0055	.	.	.0438	.0042		
**3**	**.4200**	.1214	.0165	.	.0183	.	.0002		
**3**	**.3689**	.1234	.0285	.	.0298	.0513	.		
**3**	**.4071**	.0530	.0220	.0312	.	.	.0142		
**3**	**.3826**	.0813	.0131	.0160	.	.0387	.		
**3**	**.4137**	.0701	.0172	.0065	.0076	.	.		
**3**	**.4719**	.	.	.	.0025	.0221	.0031		
**3**	**.4403**	.	.	.0341	.	.0052	.0235		
**3**	**.4432**	.	.	.0508	.0023	.	.0314		
**3**	**.4613**	.	.	.0137	.0025	.0134	.		
**3**	**.4526**	.	.0218	.	.	.0555	.0003		
**3**	**.4873**	.	.0066	.	.0207	.	.0048		
**3**	**.4486**	.	.0263	.	.0042	.0435	.		
**3**	**.4455**	.	.0000	.0626	.	.	.0404		
**3**	**.4496**	.	.0142	.0032	.	.0362	.		
**3**	**.4716**	.	.0030	.0205	.0142	.	.		
**3**	**.3994**	.0750	.	.	.	.0398	.0065		
**3**	**.4364**	.0576	.	.	.0029	.	.0020		
**3**	**.3352**	.1398	.	.	.0707	.1032	.		
**3**	**.4170**	.0285	.	.0223	.	.	.0143		
**3**	**.3838**	.0800	.	.0221	.	.0474	.		
**3**	**.4288**	.0458	.	.0096	.0025	.	.		
**3**	**.4151**	.0930	.0241	.	.	.	.0005		
**3**	**.3947**	.0582	.0112	.	.	.0209	.		
**3**	**.4135**	.0787	.0249	.	.0021	.	.		
**3**	**.4129**	.0730	.0184	.0027	.	.	.		
**4**	**.4916**	.	.	.	.0042	.0845	.0011		
**4**	**.4536**	.	.	.0422	.	.0127	.0118		
**4**	**.4531**	.	.	.1230	.0132	.	.0334		
**4**	**.4651**	.	.	.0275	.0003	.0214	.		

**4**	**.4954**	.	.0004	.	.	.0462	.0013		
**4**	**.5413**	.	.0348	.	.0002	.	.0071		
**4**	**.4922**	.	.0004	.	.0044	.0561	.		
**4**	**.4600**	.	.0063	.0815	.	.	.0287		
**4**	**.4640**	.	.0015	.0327	.	.0248	.		
**4**	**.4838**	.	.0027	.0645	.0049	.	.		
**4**	**.4000**	.0958	.	.	.	.0426	.0067		
**4**	**.4364**	.1397	.	.	.0062	.	.0021		
**4**	**.3973**	.0953	.	.	.0093	.0412	.		
**4**	**.4291**	.0372	.	.0135	.	.	.0024		
**4**	**.3957**	.0697	.	.0109	.	.0358	.		
**4**	**.4309**	.0556	.	.0077	.0006	.	.		
**4**	**.4383**	.1033	.0043	.	.	.	.0009		
**4**	**.3986**	.0980	.0080	.	.	.0405	.		
**4**	**.4202**	.1282	.0184	.	.0190	.	.		
**4**	**.4213**	.0675	.0102	.0179	.	.	.		
**4**	**.4745**	.	.	.	.	.0354	.0025		
**4**	**.4940**	.	.	.	.0159	.	.0066		
**4**	**.4750**	.	.	.	.0020	.0256	.		
**4**	**.4455**	.	.	.0644	.	.	.0407		
**4**	**.4638**	.	.	.0132	.	.0224	.		
**4**	**.4746**	.	.	.0260	.0116	.	.		
**4**	**.5081**	.	.0018	.	.	.	.0049		
**4**	**.4529**	.	.0241	.	.	.0601	.		
**4**	**.4922**	.	.0084	.	.0208	.	.		
**4**	**.4859**	.	.0003	.0271	.	.	.		
**4**	**.4393**	.0706	.	.	.	.	.0027		
**4**	**.4059**	.0711	.	.	.	.0360	.		
**4**	**.4384**	.0622	.	.	.0035	.	.		
**4**	**.4313**	.0549	.	.0106	.	.	.		
**4**	**.4156**	.0974	.0263	.	.	.	.		
**5**	**.4958**	.	.	.	.	.0837	.0010		
**5**	**.5761**	.	.	.	.0033	.	.0033		
**5**	**.4927**	.	.	.	.0041	.0867	.		
**5**	**.4663**	.	.	.1132	.	.	.0238		

**5**	**.4655**	.	.	.0313	.	.0247	.		
**5**	**.4865**	.	.	.0929	.0036	.	.		
**5**	**.5416**	.	.0379	.	.	.	.0070		
**5**	**.4966**	.	.0001	.	.	.0519	.		
**5**	**.5484**	.	.0310	.	.0001	.	.		
**5**	**.4888**	.	.0013	.0598	.	.	.		
**5**	**.4426**	.1369	.	.	.	.	.0020		
**5**	**.4066**	.0902	.	.	.	.0380	.		
**5**	**.4386**	.1409	.	.	.0060	.	.		
**5**	**.4315**	.0586	.	.0131	.	.	.		
**5**	**.4392**	.1093	.0054	.	.	.	.		
**5**	**.5099**	.	.	.	.	.	.0059		
**5**	**.4770**	.	.	.	.	.0388	.		
**5**	**.5006**	.	.	.	.0152	.	.		
**5**	**.4862**	.	.	.0296	.	.	.		
**5**	**.5130**	.	.0028	.	.	.	.		
**5**	**.4419**	.0739	.	.	.	.	.		
**6**	**.5795**	.	.	.	.	.	.0032		
**6**	**.4968**	.	.	.	.	.0858	.		
**6**	**.5794**	.	.	.	.0032	.	.		
**6**	**.4901**	.	.	.0925	.	.	.		
**6**	**.5485**	.	.0341	.	.	.	.		
**6**	**.4446**	.1380	.	.	.	.	.		
**6**	**.5158**	.	.	.	.	.	.		
**IN**	**w_SMATT**	**w_PMD**	**w_PMV**	**w_M1**	**w_preSMA**	**w_SMA**	**w_S1**		
*****	.0822	.1464	.0488	.0846	.0645	.1061	.0500		
**0**	.3660	.4357	.2476	.3284	.3585	.4043	.2897		
**1**	.0464	.1091	.0093	.0304	.0474	.0778	.0175		
**2**	.0215	.0804	.0138	.0200	.0187	.0451	.0109		
**3**	.0180	.0768	.0138	.0267	.0107	.0368	.0117		
**4**	.0213	.0831	.0099	.0375	.0077	.0390	.0102		
**5**	.0351	.1016	.0131	.0566	.0054	.0539	.0072		
**6**	.0669	.1380	.0341	.0925	.0032	.0858	.0032		

VAR	_LABEL_	OVERALL	size0	size1	size2	size3	size4	size5	size6
**w_PMD**	**w_PMD**	.1464	.4357	.1091	.0804	.0768	.0831	.1016	.1380
**w_SMA**	**w_SMA**	.1061	.4043	.0778	.0451	.0368	.0390	.0539	.0858
**w_M1**	**w_M1**	.0846	.3284	.0304	.0200	.0267	.0375	.0566	.0925
**w_SMATT**	**w_SMATT**	.0822	.3660	.0464	.0215	.0180	.0213	.0351	.0669
**w_preSMA**	**w_preSMA**	.0645	.3585	.0474	.0187	.0107	.0077	.0054	.0032
**w_S1**	**w_S1**	.0500	.2897	.0175	.0109	.0117	.0102	.0072	.0032
**w_PMV**	**w_PMV**	.0488	.2476	.0093	.0138	.0138	.0099	.0131	.0341

You requested	1000	bootstrap samples

Note:
X_1 = w-SMATT-LL
X_2 = w-PMD-LL
X_3 = w-PMV-LL
X_4 = w-M1-LL
X_5 = w-preSMA-LL
X_6 = w-SMA-LL
X_7 = w-S1-LL

Obs	dominance	I	J	Dij	Dij_mean	Dij_se	PIJ	PJI	Pijno	reprod
1	complete	1	2	0.0	0.4865	0.087	0.002	0.029	0.969	0.029
2	complete	1	3	0.5	0.4035	0.197	0.000	0.193	0.807	0.807
3	complete	1	4	0.5	0.5020	0.045	0.006	0.002	0.992	0.992
4	complete	1	5	0.5	0.4990	0.022	0.000	0.002	0.998	0.998
5	complete	1	6	0.5	0.4940	0.054	0.000	0.012	0.988	0.988
6	complete	1	7	0.5	0.5020	0.032	0.004	0.000	0.996	0.996
7	complete	2	1	1.0	0.5135	0.087	0.029	0.002	0.969	0.029
8	complete	2	3	1.0	0.4225	0.186	0.004	0.159	0.837	0.004
9	complete	2	4	1.0	0.5105	0.085	0.025	0.004	0.971	0.025
10	complete	2	5	1.0	0.5085	0.075	0.020	0.003	0.977	0.020
11	complete	2	6	1.0	0.4970	0.059	0.004	0.010	0.986	0.004
12	complete	2	7	1.0	0.5315	0.124	0.064	0.001	0.935	0.064
13	complete	3	1	0.5	0.5965	0.197	0.193	0.000	0.807	0.807
14	complete	3	2	0.0	0.5775	0.186	0.159	0.004	0.837	0.004
15	complete	3	4	0.5	0.6245	0.220	0.252	0.003	0.745	0.745
16	complete	3	5	0.5	0.6565	0.238	0.319	0.006	0.675	0.675
17	complete	3	6	0.5	0.7505	0.251	0.502	0.001	0.497	0.497

**18**	compl ete	3	7	0.5	0.6290	0.219	0.258	0.000	0.742	0.742
**19**	compl ete	4	1	0.5	0.4980	0.045	0.002	0.006	0.992	0.992
**20**	compl ete	4	2	0.0	0.4895	0.085	0.004	0.025	0.971	0.025
**21**	compl ete	4	3	0.5	0.3755	0.220	0.003	0.252	0.745	0.745
**22**	compl ete	4	5	0.5	0.5020	0.074	0.013	0.009	0.978	0.978
**23**	compl ete	4	6	0.5	0.4760	0.118	0.005	0.053	0.942	0.942
**24**	compl ete	4	7	0.5	0.5035	0.047	0.008	0.001	0.991	0.991
**25**	compl ete	5	1	0.5	0.5010	0.022	0.002	0.000	0.998	0.998
**26**	compl ete	5	2	0.0	0.4915	0.075	0.003	0.020	0.977	0.020
**27**	compl ete	5	3	0.5	0.3435	0.238	0.006	0.319	0.675	0.675
**28**	compl ete	5	4	0.5	0.4980	0.074	0.009	0.013	0.978	0.978
**29**	compl ete	5	6	0.5	0.4870	0.097	0.006	0.032	0.962	0.962
**30**	compl ete	5	7	0.5	0.5075	0.061	0.015	0.000	0.985	0.985
**31**	compl ete	6	1	0.5	0.5060	0.054	0.012	0.000	0.988	0.988
**32**	compl ete	6	2	0.0	0.5030	0.059	0.010	0.004	0.986	0.004
**33**	compl ete	6	3	0.5	0.2495	0.251	0.001	0.502	0.497	0.497
**34**	compl ete	6	4	0.5	0.5240	0.118	0.053	0.005	0.942	0.942
**35**	compl ete	6	5	0.5	0.5130	0.097	0.032	0.006	0.962	0.962
**36**	compl ete	6	7	0.5	0.5190	0.096	0.038	0.000	0.962	0.962
**37**	compl ete	7	1	0.5	0.4980	0.032	0.000	0.004	0.996	0.996
**38**	compl ete	7	2	0.0	0.4685	0.124	0.001	0.064	0.935	0.064
**39**	compl ete	7	3	0.5	0.3710	0.219	0.000	0.258	0.742	0.742
**40**	compl ete	7	4	0.5	0.4965	0.047	0.001	0.008	0.991	0.991

**41**	complete	7	5	0.5	0.4925	0.061	0.000	0.015	0.985	0.985
**42**	complete	7	6	0.5	0.4810	0.096	0.000	0.038	0.962	0.962
**43**	conditio	1	2	0.0	0.5025	0.282	0.161	0.156	0.683	0.156
**44**	conditio	1	3	1.0	0.1755	0.246	0.007	0.656	0.337	0.007
**45**	conditio	1	4	0.5	0.6865	0.254	0.385	0.012	0.603	0.603
**46**	conditio	1	5	0.5	0.5950	0.245	0.233	0.043	0.724	0.724
**47**	conditio	1	6	0.0	0.3390	0.253	0.019	0.341	0.640	0.341
**48**	conditio	1	7	1.0	0.7365	0.253	0.476	0.003	0.521	0.476
**49**	conditio	2	1	1.0	0.4975	0.282	0.156	0.161	0.683	0.156
**50**	conditio	2	3	1.0	0.2200	0.276	0.029	0.589	0.382	0.029
**51**	conditio	2	4	1.0	0.6620	0.265	0.355	0.031	0.614	0.355
**52**	conditio	2	5	1.0	0.5815	0.240	0.210	0.047	0.743	0.210
**53**	conditio	2	6	1.0	0.3780	0.273	0.057	0.301	0.642	0.057
**54**	conditio	2	7	1.0	0.8085	0.262	0.636	0.019	0.345	0.636
**55**	conditio	3	1	0.0	0.8245	0.246	0.656	0.007	0.337	0.007
**56**	conditio	3	2	0.0	0.7800	0.276	0.589	0.029	0.382	0.029
**57**	conditio	3	4	0.0	0.8780	0.222	0.762	0.006	0.232	0.006
**58**	conditio	3	5	0.5	0.8840	0.226	0.781	0.013	0.206	0.206
**59**	conditio	3	6	0.0	0.8730	0.233	0.760	0.014	0.226	0.014
**60**	conditio	3	7	0.5	0.9095	0.198	0.823	0.004	0.173	0.173
**61**	conditio	4	1	0.5	0.3135	0.254	0.012	0.385	0.603	0.603
**62**	conditio	4	2	0.0	0.3380	0.265	0.031	0.355	0.614	0.355
**63**	conditio	4	3	1.0	0.1220	0.222	0.006	0.762	0.232	0.006

**64**	conditio	4	5	0.5	0.4805	0.173	0.041	0.080	0.879	0.879
**65**	conditio	4	6	0.5	0.2505	0.263	0.013	0.512	0.475	0.475
**66**	conditio	4	7	1.0	0.5230	0.158	0.074	0.028	0.898	0.074
**67**	conditio	5	1	0.5	0.4050	0.245	0.043	0.233	0.724	0.724
**68**	conditio	5	2	0.0	0.4185	0.240	0.047	0.210	0.743	0.210
**69**	conditio	5	3	0.5	0.1160	0.226	0.013	0.781	0.206	0.206
**70**	conditio	5	4	0.5	0.5195	0.173	0.080	0.041	0.879	0.879
**71**	conditio	5	6	0.0	0.2630	0.272	0.023	0.497	0.480	0.497
**72**	conditio	5	7	0.5	0.5720	0.231	0.189	0.045	0.766	0.766
**73**	conditio	6	1	1.0	0.6610	0.253	0.341	0.019	0.640	0.341
**74**	conditio	6	2	0.0	0.6220	0.273	0.301	0.057	0.642	0.057
**75**	conditio	6	3	1.0	0.1270	0.233	0.014	0.760	0.226	0.014
**76**	conditio	6	4	0.5	0.7495	0.263	0.512	0.013	0.475	0.475
**77**	conditio	6	5	1.0	0.7370	0.272	0.497	0.023	0.480	0.497
**78**	conditio	6	7	1.0	0.7975	0.255	0.604	0.009	0.387	0.604
**79**	conditio	7	1	0.0	0.2635	0.253	0.003	0.476	0.521	0.476
**80**	conditio	7	2	0.0	0.1915	0.262	0.019	0.636	0.345	0.636
**81**	conditio	7	3	0.5	0.0905	0.198	0.004	0.823	0.173	0.173
**82**	conditio	7	4	0.0	0.4770	0.158	0.028	0.074	0.898	0.074
**83**	conditio	7	5	0.5	0.4280	0.231	0.045	0.189	0.766	0.766
**84**	conditio	7	6	0.0	0.2025	0.255	0.009	0.604	0.387	0.604
**85**	general	1	2	0.0	0.5100	0.500	0.510	0.490	0.000	0.490
**86**	general	1	3	1.0	0.0560	0.230	0.056	0.944	0.000	0.056

**87**	general	1	4	0.0	0.9210	0.270	0.921	0.079	0.000	0.079
**88**	general	1	5	1.0	0.7000	0.458	0.700	0.300	0.000	0.700
**89**	general	1	6	0.0	0.1850	0.388	0.185	0.815	0.000	0.815
**90**	general	1	7	1.0	0.8900	0.313	0.890	0.110	0.000	0.890
**91**	general	2	1	1.0	0.4900	0.500	0.490	0.510	0.000	0.490
**92**	general	2	3	1.0	0.1180	0.323	0.118	0.882	0.000	0.118
**93**	general	2	4	1.0	0.8390	0.368	0.839	0.161	0.000	0.839
**94**	general	2	5	1.0	0.6430	0.479	0.643	0.357	0.000	0.643
**95**	general	2	6	1.0	0.2620	0.440	0.262	0.738	0.000	0.262
**96**	general	2	7	1.0	0.9090	0.288	0.909	0.091	0.000	0.909
**97**	general	3	1	0.0	0.9440	0.230	0.944	0.056	0.000	0.056
**98**	general	3	2	0.0	0.8820	0.323	0.882	0.118	0.000	0.118
**99**	general	3	4	0.0	0.9660	0.181	0.966	0.034	0.000	0.034
**100**	general	3	5	0.0	0.9590	0.198	0.959	0.041	0.000	0.041
**101**	general	3	6	0.0	0.9460	0.226	0.946	0.054	0.000	0.054
**102**	general	3	7	0.0	0.9700	0.171	0.970	0.030	0.000	0.030
**103**	general	4	1	1.0	0.0790	0.270	0.079	0.921	0.000	0.079
**104**	general	4	2	0.0	0.1610	0.368	0.161	0.839	0.000	0.839
**105**	general	4	3	1.0	0.0340	0.181	0.034	0.966	0.000	0.034
**106**	general	4	5	1.0	0.2660	0.442	0.266	0.734	0.000	0.266
**107**	general	4	6	0.0	0.0670	0.250	0.067	0.933	0.000	0.933
**108**	general	4	7	1.0	0.4890	0.500	0.489	0.511	0.000	0.489
**109**	general	5	1	0.0	0.3000	0.458	0.300	0.700	0.000	0.700

**110**	genera l	5	2	0.0	0.3570	0.479	0.357	0.643	0.000	0.643
**111**	genera l	5	3	1.0	0.0410	0.198	0.041	0.959	0.000	0.041
**112**	genera l	5	4	0.0	0.7340	0.442	0.734	0.266	0.000	0.266
**113**	genera l	5	6	0.0	0.1130	0.317	0.113	0.887	0.000	0.887
**114**	genera l	5	7	1.0	0.7110	0.454	0.711	0.289	0.000	0.711
**115**	genera l	6	1	1.0	0.8150	0.388	0.815	0.185	0.000	0.815
**116**	genera l	6	2	0.0	0.7380	0.440	0.738	0.262	0.000	0.262
**117**	genera l	6	3	1.0	0.0540	0.226	0.054	0.946	0.000	0.054
**118**	genera l	6	4	1.0	0.9330	0.250	0.933	0.067	0.000	0.933
**119**	genera l	6	5	1.0	0.8870	0.317	0.887	0.113	0.000	0.887
**120**	genera l	6	7	1.0	0.9320	0.252	0.932	0.068	0.000	0.932
**121**	genera l	7	1	0.0	0.1100	0.313	0.110	0.890	0.000	0.890
**122**	genera l	7	2	0.0	0.0910	0.288	0.091	0.909	0.000	0.909
**123**	genera l	7	3	1.0	0.0300	0.171	0.030	0.970	0.000	0.030
**124**	genera l	7	4	0.0	0.5110	0.500	0.511	0.489	0.000	0.489
**125**	genera l	7	5	0.0	0.2890	0.454	0.289	0.711	0.000	0.711
**126**	genera l	7	6	0.0	0.0680	0.252	0.068	0.932	0.000	0.932

Part IV. Goodness of fit tests for multivariable regression

Dependent variable:

Motor impairment in the chronic phase after stroke: FM-UE six months

Independent variables:

Motor impairment in the acute phase after stroke : FM-UE one week

Weighted lesion load within premotor dorsal pathway: w-PMD-LL

Age at stroke onset, dichotomised (cut-off 70 years): Age

Assumptions:

Online Table IV. Non-perfect multicollinearity

First method: Variance Inflation Factor (VIF)

Independent variables	VIF
FM-UE one week	1.418
w-PMD-LL	1.601
Age	1.162

Second method: Tolerance

Independent variables	Tolerance
FM-UE one week	.705
w-PMD-LL	.625
Age	.860

Third method: Correlation matrix (Pearson's r)

	FM-UE one week	w-PMD-LL	Age
FM-UE one week	1	-.541	-.162
w-PMD-LL	-.541	1	.371
Age	-.162	.371	1

Online Figure II. Homoscedasticity

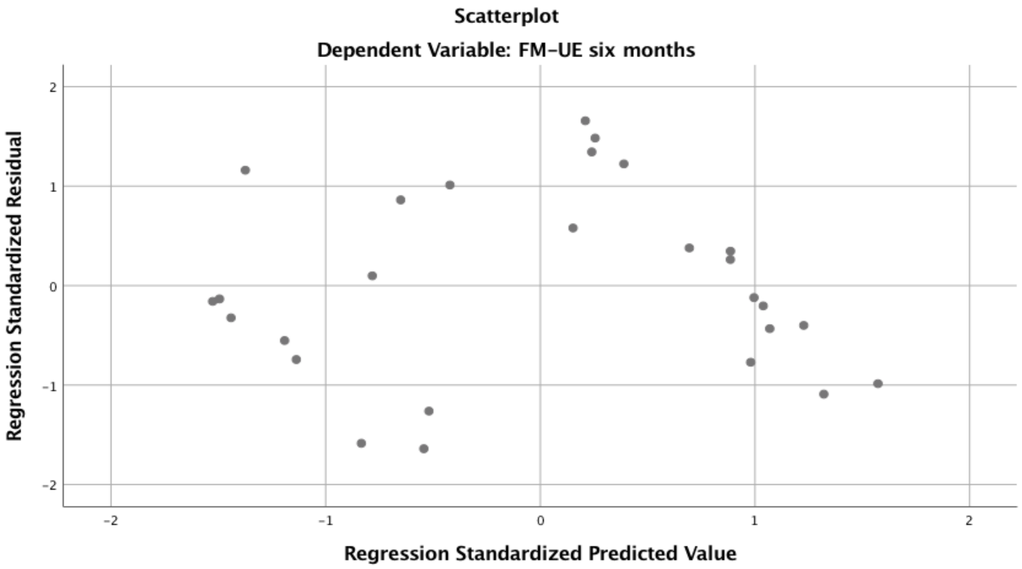

Method: visual inspection scatterplot

Online Figure III. Normally distributed errors – visual inspection histogram

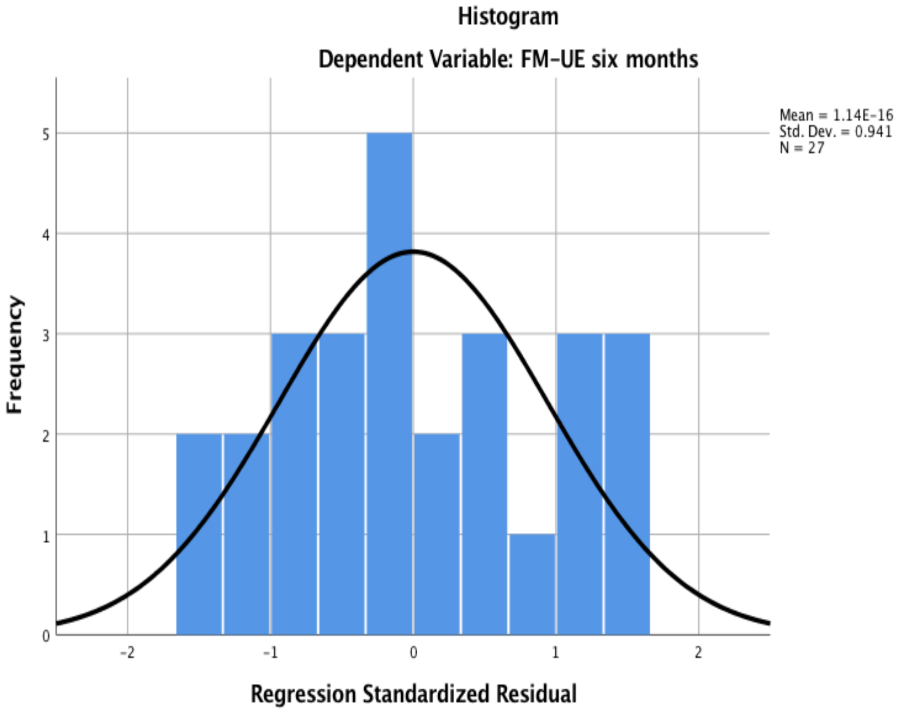

Online Figure IV. Normally distributed errors – Normal P-Plots

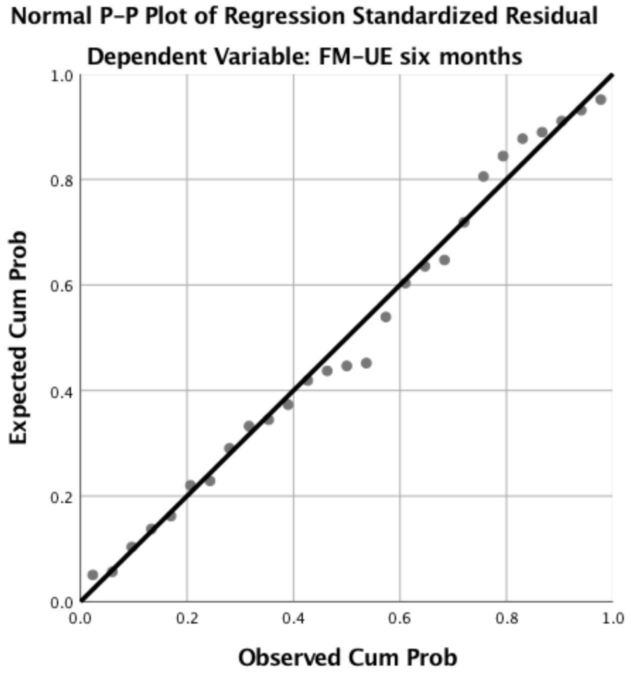

Kolmogorov Smirnov test for normally distributed errors

Variable: Standardised residuals
Results: statistic = .154; degrees of freedom = 27; p = .099

Fourth method: Shapiro-Wilk test
Results: statistic = .962; degrees of freedom = 27; p = .415

Independent errors

Method: Durbin-Watson test = 1.375

Cross-validation

Method: Adjusted R_2 = .785

Online Table V. Resampling – Bootstrapping

	B	SE B	95% CI lower bound		p
			Lower	Upper	
FM-UE one week	.684	.121	.442	.910	.001
w-PMD-LL	-.010	.003	-.014	-.003	.008
Age	13.706	5.357	2.467	23.654	.026

(based on 1000 bootstrap samples)

Part V. Differences in weighted lesion load within CST sub-pathways

Online Figure V. Lesion load within CST pathways

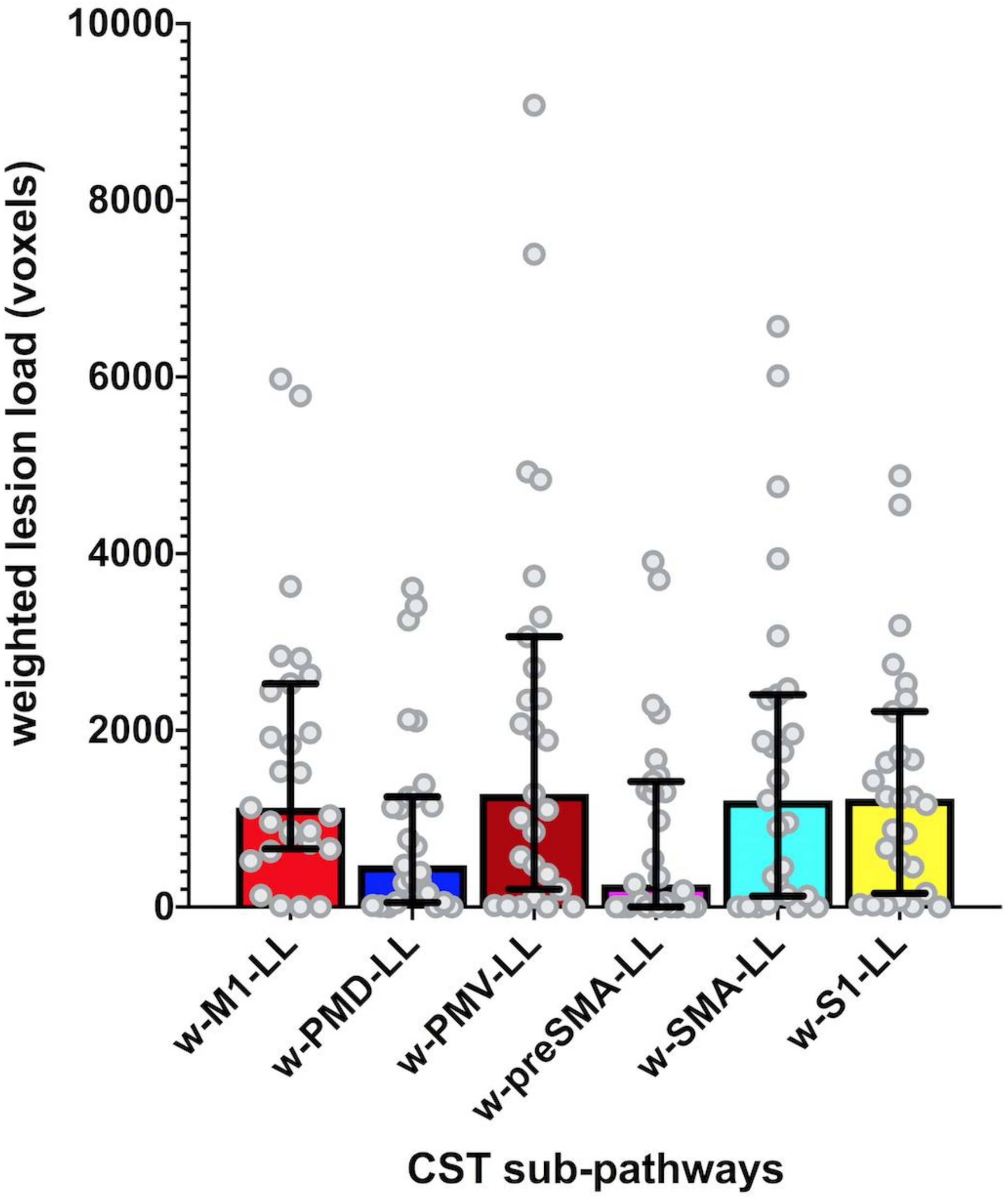

Each dot represents individual lesion load within each CST sub-pathway; boxplots report median and bars refer to interquartile range.

Online Table VI. Descriptive statistics

	Mean	SD	Q1	Q2	Q3
w-PMD-LL	942	1088	51	473	1247
w-M1-LL	1695	1553	658	1125	2526
w-PMV-LL	2062	2317	201	1280	3060
w-preSMA-LL	852	1127	0	257	1419
w-SMA-LL	1662	1853	123	1209	2401
w-S1-LL	1387	1330	154	1228	2213

First step: non-parametric repeated measure

Method: Friedman test

Results: Weighted lesion load was significant different between CST sub-pathways, $\chi_2(5)$ = 38.21, *p* <.001.

Online Table VII. Friedman test

	Mean Rank
w-PMD-LL	2.52
w-M1-LL	4.52
w-PMV-LL	4.28
w-preSMA-LL	2.11
w-SMA-LL	4.06
w-S1-LL	3.52

Second step: non-parametric related group comparisons

Method: Wilcoxon signed rank test

Results: Wilcoxon signed rank test was used to further investigate results from Friedman test. To this end, a Bonferroni correction was applied and so all effects are reported at a .003 level of significance. Z score, effect size and significance (Exact test, 2-tailed) are reported in the table below for each comparison.

Online Table VIII. Wilcoxon signed rank test

First variable	Second variable	Z	Effect size (r)	p
w-PMD-LL	w-M1-LL	-3.46	-.48	.000*
w-PMD-LL	w-PMV-LL	-3.39	-.47	.000*
w-PMD-LL	w-preSMA-LL	1.01	.14	.325
w-PMD-LL	w-SMA-LL	-3.75	-.52	.000*
w-PMD-LL	w-S1-LL	-3.01	-.42	.001*
w-M1-LL	w-PMV-LL	-0.93	-.13	.367
w-M1-LL	w-preSMA-LL	3.52	.49	.000*
w-M1-LL	w-SMA-LL	0.67	.09	.515
w-M1-LL	w-S1-LL	3.39	.47	.000*
w-PMV-LL	w-preSMA-LL	3.62	.50	.000*
w-PMV-LL	w-SMA-LL	1.64	.23	.105
w-PMV-LL	w-S1-LL	1.66	.23	.099
w-preSMA-LL	w-SMA-LL	-3.66	-.51	.000*
w-preSMA-LL	w-S1-LL	-2.91	-.40	.003
w-SMA-LL	w-S1-LL	1.08	.15	.290

Level of significance based on Bonferroni correction (.05/15=.003)
